# Heparin-induced thrombocytopaenia presenting as acute aortic mural thrombosis

**DOI:** 10.1259/bjrcr.20180025

**Published:** 2018-10-05

**Authors:** Maya Joanne Bienz, Pawel Obrocki, James Russell, Rajesh Jena, Iosif Alexandru Mendichovszky

**Affiliations:** 1 Department of Oncology, Cambridge University Hospitals NHS Foundation Trust, Cambridge Biomedical Campus, Cambridge, UK; 2 Department of Haematology, Cambridge University Hospitals NHS Foundation Trust, Cambridge Biomedical Campus, Cambridge, UK; 3 Department of Radiology, Cambridge University Hospitals NHS Foundation Trust, Cambridge Biomedical Campus, Cambridge, UK

## Abstract

Heparin-induced thrombocytopaenia (HIT) is a life and limb-threatening acquired autoimmune complication of heparin-based treatment, characterised by thrombocytopaenia and thrombosis. We present a case of a 77-year-old female with concomitant metastatic ovarian and breast cancer who presented to our institution with worsening shortness of breath. She had been diagnosed with acute pulmonary embolism 1 month earlier that was treated with therapeutic low molecular weight heparin (LMWH). In view of her worsening symptoms, CT imaging was performed. This demonstrated significant progression of the bilateral pulmonary emboli and new mural thrombosis of the thoracic aorta, despite being compliant with therapeutic anticoagulation. She had also developed thrombocytopaenia since commencing LMWH, which raised the clinical suspicion of HIT syndrome. The HIT pre-test probability score was intermediate and LMWH was immediately discontinued pending further investigation. She was commenced on rivaroxaban, a direct oral anticoagulant, and her platelet count soon recovered. Laboratory testing was strongly positive on both immunological and functional assays, thus confirming a diagnosis of HIT syndrome. A repeat CT scan 3 weeks later showed a reduction in the overall thrombus load. Whilst venous thrombosis is observed in as many as half of patients with HIT, arterial thrombosis is a far less common event. Furthermore, arterial involvement usually affects the distal vessels with significant atherosclerotic burden and typically presents as acute limb ischaemia or ischaemic stroke. Aortic thrombosis, as in this case, is a rare complication of HIT syndrome.

## Clinical presentation

**Figure 1.**
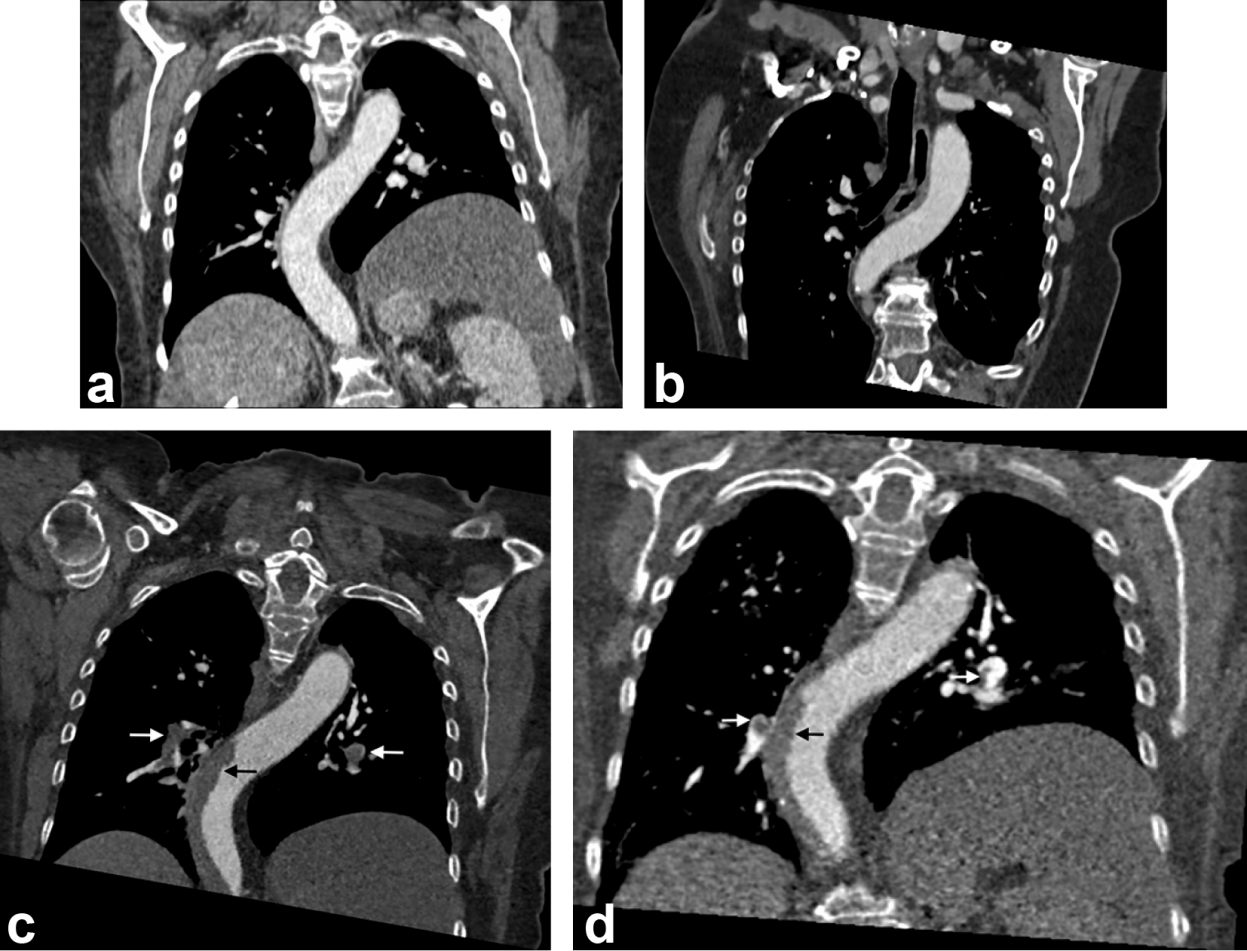
(a) Coronal CT image 2 months prior to admission showing no evidence of thrombosis in the thoracic aorta. (b) Staging CT 1 month prior to admission showing no evidence of thrombosis in the thoracic aorta. (c) Coronal CT image at the time of admission showing acute mural nonocclusive thoracic aortic thrombus (white arrow, pulmonary emboli; black arrow, aortic thrombosis). (d) Coronal CT image 3 weeks after commencement of rivaroxaban showing moderate reduction in aortic thrombosis and significant reduction of embolic burden (white arrow, pulmonary emboli; black arrow, aortic thrombosis).

**Figure 2.**
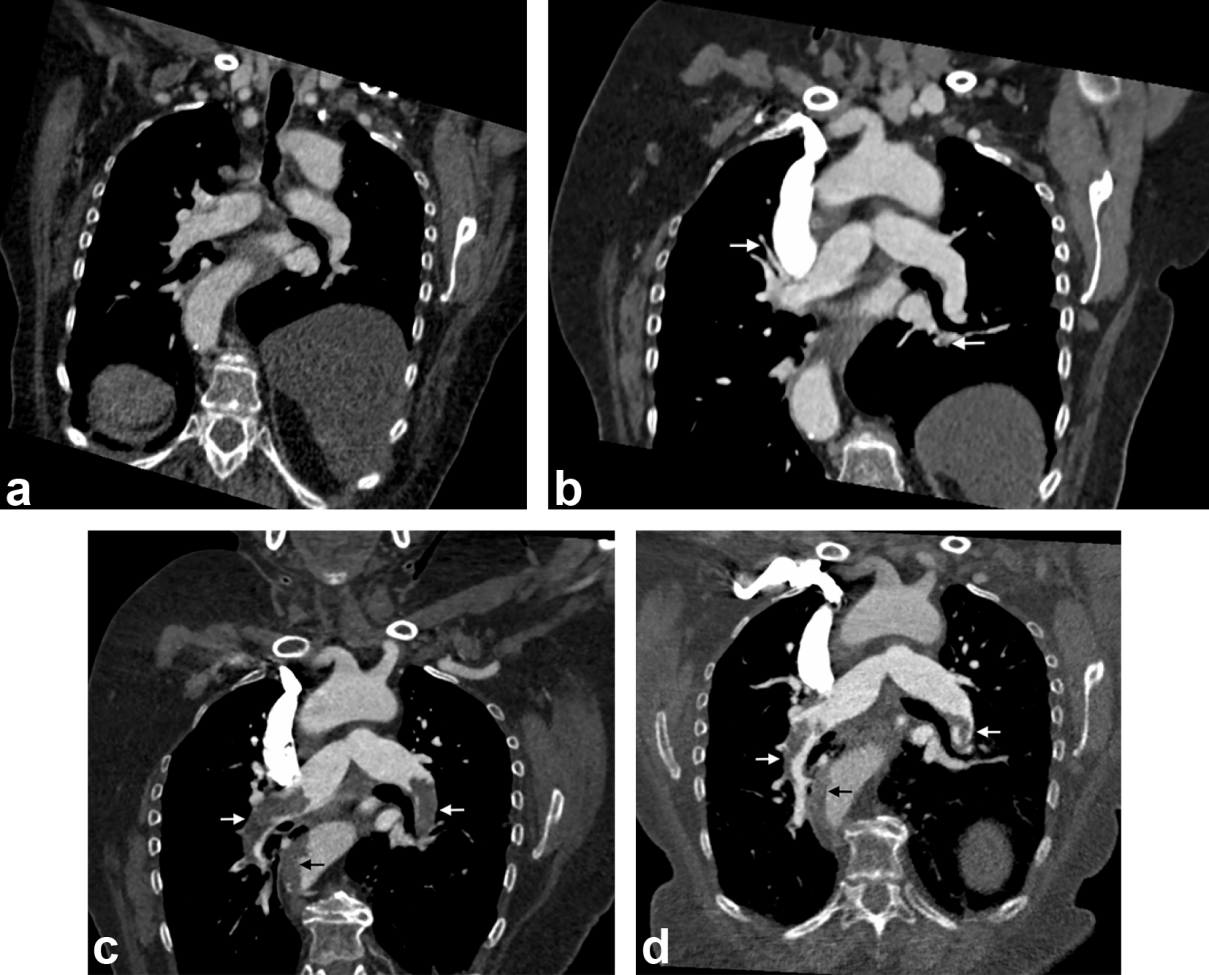
(a) Coronal CT image 2 months prior to admission showing no evidence of thrombosis in the pulmonary vasculature. (b) Staging CT 1 month prior to admission showing bilateral pulmonary emboli but patent main pulmonary arteries. (c) Coronal CT image at the time of admission showing new extensive pulmonary emboli within the main pulmonary arteries bilaterally and new aortic thrombosis (white arrow, pulmonary emboli; black arrow, aortic thrombosis). (d) Coronal CT image 3 weeks after commencement of rivaroxaban showing reduction in volume of thrombus within main pulmonary arteries and aorta (white arrow, pulmonary emboli; black arrow, aortic thrombosis).

A 77-year-old female presented to our cancer assessment unit with worsening exertional shortness of breath. Physical examination of the chest was unremarkable; however, she was hypoxic, with pulse-oximetry measurements of 84% on 2L of oxygen per minute. The rest of her observations were stable. She had a history of metastatic breast cancer treated with letrozole (hormone therapy) and metastatic high-grade serous ovarian carcinoma for which she had received six cycles of carboplatin chemotherapy. 1 month earlier a routine CT staging examination had shown stable malignant disease, but it also found new bilateral lower lobe pulmonary emboli when compared to previous imaging (Figures 1a, b, 2a and b) and thrombosis of the left common femoral vein. She was managed with once daily therapeutic-dose LMWH (dalteparin).

## Diagnostic imaging and laboratory investigations

On admission, a repeat CT thorax, abdomen and pelvis showed progression of the previously identified pulmonary emboli, with new thrombi seen within the left and right main pulmonary arteries (Figure 1c), but with no radiological features to suggest right heart strain. New mural hypodensities were also identified in the thoracic and upper abdominal aorta in keeping with extensive acute non-occlusive mural thrombi (Figure 2c). Overall, CT imaging was strongly suggestive of a prothrombotic state, despite the patient receiving therapeutic-dose dalteparin.

The staging CT examinations were set up to opacity the thoracic aorta whilst the CTPA studies focussed on the enhancement of the pulmonary arterial tree. In both cases, the pulmonary arterial tree and aorta became well-opacified (despite the different CT protocols) and this was likely due to a combination of the patient’s circulatory status, contrast volume and injection rate, as well as the speed of the CT scanner.

**Figure 3.**
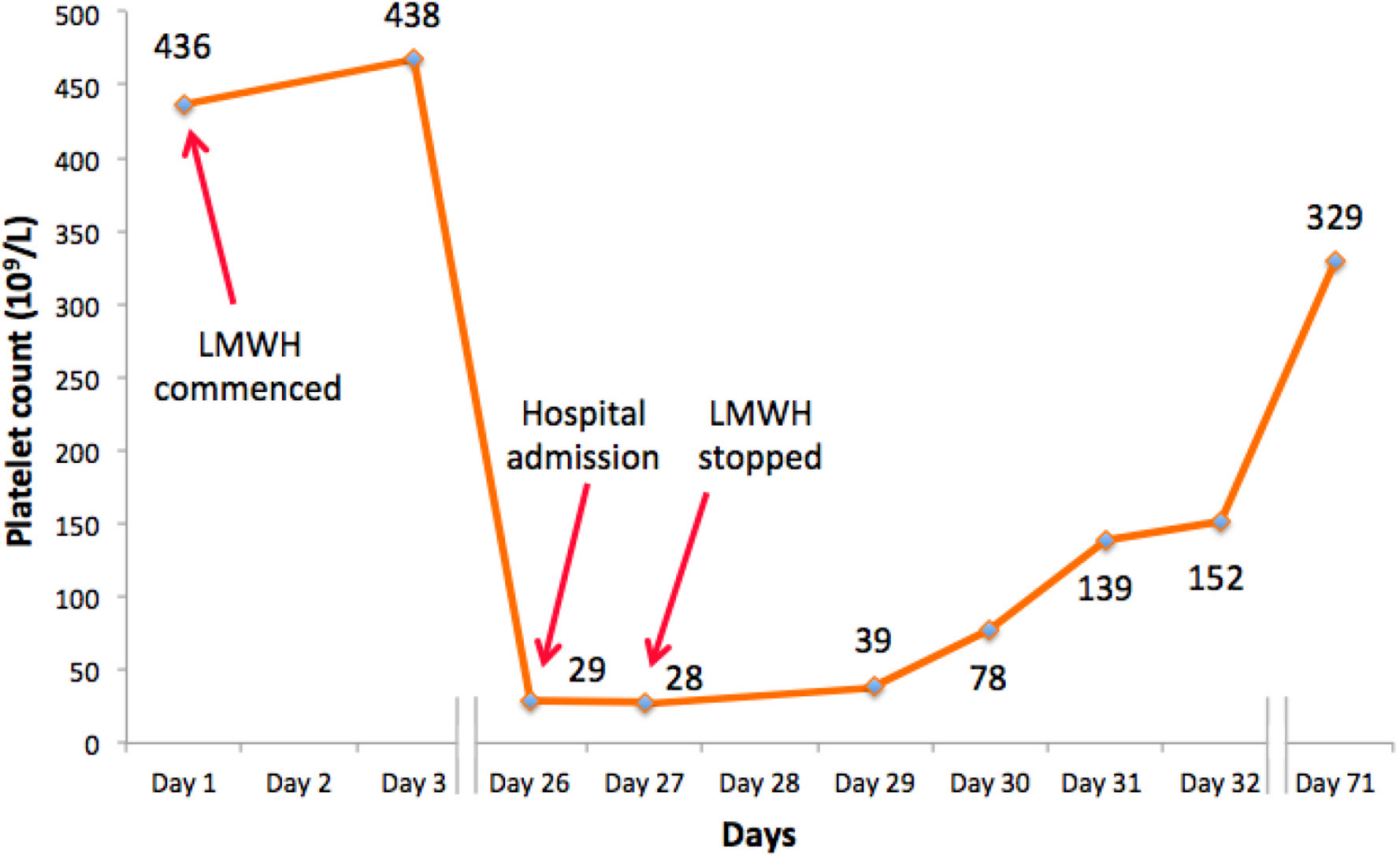
Serum platelet count recorded before, during and after the patient’s admission (x10^9^/l)

On her acute admission, the patient’s platelet count was 29 × 10^9^ l^–1^ (reference 160–370 × 10^9^ l^–1^), having been normal 3 weeks previously (468 × 10^9^ l^–1^) (Figure 3). The remainder of her blood test results were stable (Table 1). A blood film showed some platelet clumping and fibrin stranding, nonetheless multiple repeat platelet count measurements confirmed a genuine thrombocytopaenia.

**Table 1.** Blood test results on admission

White cell count	6.5 (3.6–10.5 x 10^9 ^l^–1^)	Sodium	136 (133–146 mmol l^−1^)
Haemoglobin	90 (118–158 g l^−1^)	Potassium	4.4 (3.5–5.3 mmol l^−1^)
Platelet count	29 (160–370 x 10^9 ^l^–1^)	Urea	9.8 (2.5–7.8 mmol l^−1^)
Mean platelet volume	12.6 (fl)	Creatinine	119 (44–97 μmol l^−1^)
Lymphocyte count	1.20 (1.10–4.00 x 10^9 ^l^–1^)	Adjusted calcium	2.31 (2.20–2.60 mmol l^−1^)
Basophil count	0.02 (0.00–0.20 x 10^9 ^l^–1^)	Phosphate	0.94 (0.80–1.50 mmol l^−1^)
Eosinophil count	0.14 (0.02–0.50 x 10^9^ l^–1^)	Magnesium	0.73 (0.70–1.00 mmol l^−1^)
Monocyte count	0.34 (0.10–0.90 x 10^9^ l^–1^)	Alkaline phosphatase	82 (30–130 U l^−1^)
Neutrophil count	4.67 (1.50–7.70 x 10^9^ l^–1^)	Albumin	32 (35–50 g l^−1^)
CRP	21 (0–6 mg l^−1^)	Alanine transaminase	23 (7–40 U l^−1^)
Lactate	1.3 (0.6–1.4 mmol l^−1^)	Total bilirubin	5 (0–20 μmol l^−1^)
eGFR	38 (ml^–1^ min^–1^ 1.73 m^2^)	Glucose	7.5 (3.5–5.3 mmol l^−1^)

GFR, glomerular filtration rate;

Her “4Ts” HIT score (a scoring system that estimates the pre-test probability of HIT), was intermediate at 4.^1^ Subsequent immunological HIT screening was strongly positive for immunoglobulin G anti-PF4-heparin antibodies and functional testing (Heparin-Induced Activation Test) was also positive, confirming the diagnosis of HIT. 

## Differential diagnoses

The broad differential diagnoses of her thrombocytopaenia included sepsis, chemotherapy-induced bone marrow suppression, metastatic infiltration of the bone marrow, drug-induced or immune thrombocytopaenic purpura, haematinic-deficiency and HIT. Additional causes of thrombocytopenia such as liver disease, hypersplenism, microangiopathic haemolysis (*e.g.* disseminated intavasicular coagulopathy, thrombotic thrombocytopenic purpura and haemolytic uraemic syndrome) and B12/folate deficiencies were considered, but deemed less likely.

In this case, chemotherapy-induced bone marrow suppression was less likely since the last cycle of chemotherapy had been administered 6 weeks prior to this admission and the patient had sustained a normal platelet count throughout her treatment. This new thrombocytopaenia, in the context of active LMWH therapy and progressive pulmonary and aortic thrombosis, triggered a suspicion of HIT.

A prothrombotic malignancy-related cause for the patient’s progressive arterial and venous thrombosis had been considered, but the worsening venous and new arterial thrombi following the administration of low molecular weight heparin treatment is considered a very rare entity in the context of malignancy. This again pointed to HIT being a more likely cause and this diagnosis was confirmed by immunological and functional testing for HIT, with platelet counts improving once heparin treatment was discontinued.

## Treatment and outcome

As soon as a diagnosis of HIT was suspected, dalteparin was immediately discontinued and the patient was commenced on rivaroxaban, a direct oral anticoagulant. Anti-cancer therapy was also withheld. She remained oxygen-dependent, but clinically well, and her platelet count normalised after 5 days (Figure 3). She was discharged with home oxygen and 5 weeks later her platelet count remained stable. A CTPA 3 weeks after leaving hospital showed a reduction in the overall thrombus burden within the aorta and bilateral lower lobe pulmonary arteries (Figures 1d and 2d).

## Discussion

HIT is an immune-mediated therapeutic complication of heparin-based drugs that carries significant morbidity and mortality.^2^ The hallmark of this condition is the development of thrombocytopaenia with a marked pro-thrombotic tendency following the commencement of heparin. Two different types of HIT are recognised:

- HIT Type I results from the direct action of heparin on platelets leading to platelet aggregation.^3^ This reaction occurs within the first three days of heparin administration, is short-lived, and rarely clinically significant.^3^ The associated thrombocytopaenia tends to be mild (100–150 × 10^9^ l^–1^), resolves spontaneously and there is no associated increase in risk of thrombosis.^3,4^
- HIT Type II is an antibody-driven immune reaction in response to heparin administration, which typically occurs 4–14 days after exposure to heparin.^4^ This results in a significant fall in platelet count (usually >50% of baseline) and an increased risk of both venous and arterial thrombosis.^4^ The rest of this report will focus on this HIT type.

Development of anti-platelet factor 4 (PF4) antibodies is a central mechanism in the pathophysiology of HIT. PF4 is a pro-coagulant protein contained within the alpha granules of megakaryocytes and plays a role in coagulation and inflammation.^4^ Additionally, PF4 can neutralise the effects of heparin.^5^ In HIT, immunoglobulin G autoantibodies form against heparin–PF4 immune complexes that are present on the platelet surface.^4^ This stimulates platelets to secrete pro-coagulant microparticles which, in turn, have downstream effects on driving thrombin activation and induction of other inflammatory cell lines, such as monocytes.^3,4^ The result of this immune-mediated activation is a hypercoagulable state, which can precipitate catastrophic thrombosis.^4^ Simultaneously, the activated platelets are removed by macrophages in the reticuloendothelial system, causing the thrombocytopaenia present in HIT.^5^

The incidence of HIT varies considerably depending on patient and drug-related factors and may be as high as 5%.^4^ HIT is more common in patients treated with unfractionated heparin compared with LMWH.^6^ The risk is also higher if therapeutic doses are administered.^7^ Additionally, the observed incidence of HIT is higher in both female and surgical patients.^2^

As many as 55% of HIT patients will develop venous thrombosis, most commonly presenting as deep venous thrombosis or pulmonary embolism.^8^ The risk of arterial thrombosis is also elevated and ranges from 7 to 14%.^5^ Arterial thrombosis tends to occur in vessels with a significant degree of atherosclerosis, leading to limb ischaemia, myocardial infarction or stroke.^4^ Widespread aortic thrombosis involving the thoracic aorta, as described in our case, is an uncommon finding in an anatomically normal aorta and is associated with the pro-thrombotic state seen in malignancies or specific clotting factor deficiencies.^9^ Very rarely, a primary malignancy of great vessels, such as aortic angiosarcoma, can also present with aortic occlusive disease.^10^ Aortic thrombosis as a direct complication of HIT is even more rare and has previously been reported in patients who underwent surgical procedures, such as aortic root replacement or insertion of an automatic cardioverter–defibrillator.^11,12^

Other presentations of HIT include skin necrosis at the site of heparin injection, limb gangrene, central venous thrombosis or spinal ischaemia.^5^ Bleeding complications are rare in HIT despite thrombocytopaenia, and current evidence does not support platelet transfusion due to a markedly elevated risk of thrombosis.^5^

The possibility of HIT should be considered in all patients with thrombocytopaenia who have received at least four days of unfractionated heparin or LMWH. Additionally, HIT should be suspected in patients who present with thrombosis and have been exposed to heparin in the last 100 days.^2^ HIT cannot be confirmed based solely on laboratory testing and the diagnosis of HIT is reached by combining specific clinical criteria with positive laboratory testing.

Clinical scoring systems can be used to risk stratify a patient with suspected HIT, such as the commonly used Warkentin (4Ts) score.^1^ This score considers the following factors: the degree of thrombocytopaenia, the timing of the platelet count reduction, the presence of thrombosis or other complications of HIT, and other causes of thrombocytopaenia.^1^ Serological testing should be performed in patients with at least a moderate clinical probability of HIT.^2^

The most widely available laboratory test used to confirm HIT is the ELISA heparin/platelet factor immunoassay, which is very sensitive (99%), but has a high false-positive rate, limiting its utility in low risk patients.^13^ Functional washed platelet assays, such as serotonin release assay or heparin induced aggregation assays have a better specificity, but are only available in specialist centres and thus can cause unacceptable delay in treatment.^4^ In our patient we used the Acustar Assay (IL, Werfen, Warrington UK) for screening and the HIPA test performed at the University of Greifswald, Germany for confirmation.

In patients with a high probability of HIT, all forms of heparin, including heparin flushes, should be discontinued immediately. The pro-thrombotic state continues after cessation of heparin therapy due to circulating anti-PF4 antibodies and an alternative non-heparin-based anticoagulant should be commenced to counteract this effect.^4^ Agents used for the treatment of HIT include direct thrombin inhibitors, such as argatroban, bivalirudin or danaparoid.^4^ Direct oral anticoagulants are also considered safe and effective in the treatment of HIT.^14^ Warfarin alone should be avoided as initial therapy in patients with HIT, as it causes a rapid fall in Protein C levels which can precipitate venous limb gangrene.^2,5^ Additionally, prothrombin complex concentrate used for reversal of bleeding after warfarin use is contraindicated in patients with HIT, due to small amounts of heparin being present in certain prothrombin complex concentrate formulations.^15^

Untreated, HIT with evidence of thrombosis carries a mortality rate of up to 30%, which can be significantly reduced with prompt recognition and appropriate interventions.^3^ Platelet count usually recovers within 1 week after withdrawal of heparin and anti-PF4 antibodies typically become negative after 3–4 months.^2^ However, all patients who have experienced HIT in the past are at a higher risk of developing the complication again, thus future exposure to heparin-based treatments should be avoided.^2^

## Learning points

1. HIT is an immune-mediated complication of treatment with heparin-based therapies, characterised by thrombocytopaenia and thrombosis.
2. HIT must always be considered in the differential diagnosis for an apparent pro-thrombotic state seen on CT imaging.
3. Extensive aortic thrombosis in HIT, as presented in our case, illustrates a rare complication of this potentially life-threatening condition.
4. Direct oral anticoagulants are seemingly a safe and effective treatment choice in HIT.
5. This case highlights the importance of regular platelet count monitoring in patients receiving heparin therapy.
